# The suitability of the GERDyzer instrument in pH-test-proven laryngopharyngeal reflux patients

**DOI:** 10.1097/MD.0000000000004439

**Published:** 2016-08-07

**Authors:** Cheng-Pin Wu, Wen-Miin Liang, Chen-Chi Wang, Chi-Sen Chang, Hong-Zen Yeh, Jeng-Yuan Hsu, Chung-Wang Ko, Shou-Wu Lee, Shu-Chuan Chang, Fung-Chang Sung, Han-Chung Lien

**Affiliations:** a Health Examination Center, China Medical University Hospital, Taichung, Taiwan; b Preventive Medicine Center, China Medical University Hospital, Taichung, Taiwan; c Graduate Institute of Biostatistics, China Medical University, Taichung, Taiwan; d Department of Otolaryngology, Taichung Veterans General Hospital, Taichung, Taiwan; e School of Medicine, National Yang-Ming University, Taipei, Taiwan; f School of Speech Language Pathology & Audiology, Chung Shan Medical University, Taichung, Taiwan; g Division of Gastroenterology, Taichung Veterans General Hospital, Taichung, Taiwan; h Department of Internal Medicine, Chung Shan Medical University, Taichung, Taiwan; i Department of Internal Medicine, National Yang-Ming University, Taipei, Taiwan; j Division of Chest Medicine, Taichung Veterans General Hospital, Taichung, Taiwan; k Department of Nursing, Central Taiwan University of Science and Technology, Taichung, Taiwan; l Management Office for Health Data, China Medical University and Hospital, Taichung, Taiwan; m Institute of Clinical Medical Science, China Medical University College of Medicine, Taichung, Taiwan.

**Keywords:** GERD, LPR, PRO

## Abstract

The use of validated patient-reported outcome (PRO) instruments for the treatment outcome measure of laryngopharyngeal reflux (LPR) is crucial given the lack of objective markers. However, current symptom-based PRO instruments can only partially capture the impact of LPR. The GERD Analyzer (GERDyzer), an existing disease-specific PRO instrument, which measures multidimensional health-related quality of life (HRQL) affected by the illness rather than by any specific symptoms, has been validated in patients with erosive gastroesophageal reflux disease (GERD). Following the U.S. Food and Drug Administration PRO guidance, we cross-culturally adapted the GERDyzer instrument into Chinese, and examined the qualitative and quantitative psychometric properties of the Chinese version GERDyzer in pH-test-proven LPR patients.

The GERDyzer comprises 10 dimensions of HRQL, including general well-being, pain/discomfort, physical health, diet, energy, activities, leisure activities, social life, mood, and sleep. To examine the content validity, we recruited 26 pH-test-proven LPR participants to conduct 4 focus group meetings for direct patient input on clinical manifestations and HRQL impacts. We also tested the quantitative psychometric properties, including reliability, validity, and responsiveness in 100 pH-test-proven LPR patients.

Saturation of concept elicitation was achieved from the 4 focus groups, and a strong conceptual match was evident between the GERDyzer contents and responses from the focus group participants. Cognitive debriefing assessment showed that the Chinese version GERDyzer was adequate for use by patients as it demonstrated linguistic validation and cultural harmonization. Quantitative psychometric properties showed evidence of high internal consistency (Cronbach α: 0.96), good to excellent test-retest reliability (intraclass correlation coefficient: 0.84–0.98). Confirmatory factor analysis supported a 2-factor structure. Convergent validity was confirmed by moderate correlation assessments referencing the Reflux Symptoms Index and the Reflux Questionnaire. The discriminant validity was supported by the ability to discriminate moderate-to-severe disease from mild disease. The responsiveness was also high in participants with and without typical GERD symptoms (effect sizes 1.20 and 1.21, respectively).

In conclusion, the Chinese version GERDyzer instrument is a reliable, valid, and responsive instrument for assessing HRQL in Taiwanese patients with LPR.

## Abstract 2

Supplemental Digital Content is available in the text

## 1 Introduction

Laryngopharyngeal reflux (LPR) is an extraesophageal manifestation of gastroesophageal reflux disease (GERD).^[1]^ Unlike typical GERD, the symptoms of LPR are diverse and nonspecific, and include hoarseness, globus, throat clearing, cough, and sore throat. Owing to the lack of reliable objective markers of LPR, evaluation of therapies must therefore rely on patient-reported outcome (PRO) instruments.^[2]^ Current PRO instruments of LPR-specific studies have been developed predominantly based on laryngeal symptoms.^[3–6]^ However, a symptom-based PRO instrument alone (either GERD specific or LPR specific) not only failed to capture the full range of potential reflux symptoms,^[7]^ but also did not necessarily reflect the health-related quality of life (HRQL) in patients with LPR.

According to the European PRO guidance, HRQL is a multidimensional (physical, mental, and social well-being) construct, rather than solely a list of adverse events or a scale of bothering symptoms.^[8]^ The GERD Analyzer (GERDyzer) is a PRO instrument which has been validated in patients with erosive GERD and translated into multiple languages.^[9]^ The instrument measures multidimensional HRQL that is affected by the illness rather than by any specific symptoms. Recent data have shown that 46% to 89% of patients with LPR also had subtle or obvious typical GERD symptoms.^[10,11]^ Based on the assumption that LPR and GERD are diseases with a spectrum of overlapping symptoms, the GERDyzer may allow clinicians to use only one PRO instrument to measure HRQL outcome in patients with GERD, LPR, or the overlapping of both.

To apply an existing PRO instrument in a different target population, the U.S. Food and Drug Administration (FDA) guidance emphasized the importance of including documentation of content validity of patient input by focus groups and evaluation of patient understanding by cognitive interviewing.^[12]^ Thus, we cross-culturally adapted the GERDyzer instrument into the Chinese language, and examined the content validity of the GERDyzer using focus groups in patients with LPR. We also conducted a quantitative psychometric validation study to assess the reliability, validity, and responsiveness of the GERDyzer in a sample of pH-test-proven LPR patients.

## 2 Methods

### 2.1 Study design, settings, and participants

This study used a methodological research design, which included translation of the English GERDyzer into Chinese, focus group discussion to evaluate content validity of the Chinese version GERDyzer, and assessment of psychometric properties including reliability, validity, and responsiveness. Patients with suspected LPR underwent upper gastrointestinal endoscopy, laryngoscopy, and 24-hour ambulatory pH test to assess the eligibility at baseline. The inclusion criteria required patients to be ≥20 years of age and the presence of one or more laryngeal symptoms as the primary symptoms for ≥3 months; laryngoscopic signs indicative of reflux such as posterior laryngitis, erythema, or edema of the larynx; and presence of abnormal acid exposure using 24-hour ambulatory simultaneous esophageal and pharyngeal pH monitoring when off any antisecretory drugs.^[13]^ The above techniques have been previously described in detail.^[14]^ We adopted a composite pH parameter incorporating both pathological refluxes in hypopharynx and esophagus for patient selection^[15]^ because the mechanisms of LPR involve both the microaspiration of refluxate to the airway and a vagal-mediated esophagobronchial reflex.^[16]^ Patients were excluded if any common etiologies of chronic laryngitis other than reflux existed (Supplementary Content 1). The presence of typical GERD symptoms was defined by the presence of mild symptoms of heartburn and/or regurgitation occurring at least twice a week, or moderate/severe symptoms using a modified international GERD instrument.^[17]^ The study was conducted at the Voice & Laryngeal Pathology Laboratory and Gastrointestinal Physiology & Motility Laboratory in Taichung Veterans General Hospital, Taiwan, between March 2011 and December 2015. The protocol was approved by Taichung Veterans General Hospital Institutional Review Board (#C06254-3). All participants had signed an informed consent form before the study.

### 2.2 Process of the translation of the GERDyzer

Linguistic translation of the GERDyzer instrument followed a forward-backward procedure. Two bilingual translators independently translated the GERDyzer into a Chinese version. One was translated by a senior gastroenterologist (H.-C. Lien) who was informed of the intent of the original instrument,^[18]^ and the other was translated by a bicultural and bilingual translator (S.P. Lien) without knowledge about GERD who was not informed.^[19]^ The 2 translators conducted a joint discussion to detect errors, to reconcile any discrepant interpretations of items,^[20]^ and subsequently to arrive at a unified version. Subsequently, the Chinese version was back-translated into English version by a native English teacher without a medical background (A. Lee), who has been living in Taiwan for the past 20 years and speaks fluent Chinese, and who was not informed of the concepts explored.^[21]^ The backward translated English version was verified by comparing it with the original English version of the GERDyzer by a committee comprising 3 translators (H.-C. Lien, S.P. Lien, and A. Lee), a bilingual otolaryngologist (C.-C. Wang), a language professional (S. Brenda), and a methodologist (W.-M. Liang). The committee also reviewed all the forward and backward translations, and resolved any discrepancy to produce a pre-final version.

Subsequently, cognitive debriefing interviews were conducted in a sample of 10 subjects with educational levels ranging from elementary school to university from the target population to verify their understanding of the Chinese version GERDyzer. The number of cognitive interviews was determined based on the complexity of the instrument, amount and nature of revision, and the heterogeneity of the interview sample.^[22]^ An experienced interviewer (C.-P. Wu), who had received mock interview training, performed a one-on-one interview in a private room using a semistructured questionnaire (Supplementary Content 2). The interviewer conducted the interview in a friendly and sympathetic manner. The patients were asked about the meanings of the instruction and each item of the Chinese version GERDyzer, any confusion or difficulties related to words or phrases, the meanings and experiences of the recall period and response options, any experiences not covered in the questionnaire, and any comments on the questionnaire using open-ended questions.^[22]^ The cognitive interview summary (Supplementary Content 3) was documented using the comments and discussion provided by the same committee. An item-tracking matrix was used to document the changes in items and the reasons for those changes.^[22]^ The committee approved the final version which was deemed to have semantic, idiomatic, experiential, and conceptual equivalences compared with the original English version (Supplementary Content 4). The equivalence between the original and Chinese version GERDyzer was then assessed using the content validity index (CVI) method by 5 selected highly reputed bilingual GERD experts (C.-S. Chang, H.-Z. Yeh, S.-W. Lee, C.-W. Ko, and J.-Y. Hsu).^[23]^ Among them, 4 were senior gastroenterologists of whom 2 held PhDs, including 1 professor. The other was a professor of pulmonary medicine. Each expert rated the equivalence of each item on a 4-point scale, that is, 1 not equivalent, 2 somewhat equivalent, 3 quite equivalent, and 4 highly equivalent.^[24]^ For each item, the CVI was computed as the proportion of experts giving a rating of either 3 or 4; that is to say, the agreement on the equivalence of each item. An item level-CVI (I-CVI) <80% would be considered a candidate for revision. An average I-CVI across all items >90% was considered excellent content validity.^[23]^

### 2.3 PRO instruments

GERDyzer is a validated GERD-specific PRO instrument that measures HRQL in patients with erosive GERD.^[9]^ The assessment of patients was conducted using a 10-cm visual analogue scale, accompanied by a graphic. There were 10 items representing 10 dimensions of HRQL, which were clustered into 2 factors. The first included general well-being, pain/discomfort, physical health, and diet, and was weighted by 1.0. The second comprised energy, activities, leisure activities, social life, mood, and sleep, and was weighted by 0.5. The summation of the weighted score from each item constituted the total score, ranging from 0 to 70, with a higher score indicating a worse HRQL. An international validation study of the GERDyzer including Austria, Germany, and South Africa among patients with erosive GERD has found high internal consistency (Cronbach α: 0.95), high test-retest reliability (intraclass correlation coefficient [ICC]: 0.91), and logical construct validity (Spearman correlation coefficient 0.60, −0.55, and 0.70, with the Gastrointestinal Symptom Rating Scale, the Psychological General Well-Being, and the Reflux Questionnaire [ReQuest], respectively).^[9]^

Reflux Symptoms Index (RSI) is a 9-item instrument that has been validated in a sample of pH-test-proven LPR patients for the assessment of LPR symptoms.^[5]^ The scale for each item ranged from 0 (no problem) to 5 (severe problem), with a maximum total score of 45. The Chinese version RSI is in agreement with the English version,^[25]^ and has been shown to have good internal consistency (Cronbach α: 0.74) and good test-retest reliability (ICC: 0.79).^[25]^

ReQuest is a validated GERD questionnaire that is used to assess patients with erosive or nonerosive GERD.^[26]^ A long and a short version of the ReQuest exist, and the short version was used in this study. It consists of 7 dimensions and was subdivided into 2 subscales, with a higher score indicating a worse health-HRQL: the first subscale, ReQuest-GI, included the dimensions with GI complaint (acid complaints, upper abdominal/stomach complaints, lower abdominal/digestive complaints, and nausea), and the second subscale, ReQuest-WSO, included dimensions affecting the aspects of well-being (general well-being, sleep disturbances, and other complaints). ReQuest showed a high internal consistency (Cronbach α: 0.9) and a high test-retest reliability (ICC: 0.99). The high correlation between ReQuest and GERDyzer (Spearman correlation coefficient: 0.8) demonstrated construct validity in patients with GERD.^[27]^ The Chinese version of ReQuest was approved in an international multicenter trial.^[28]^

### 2.4 Conducting focus groups

We performed focus group procedures based on a literature review.^[29,30]^ Four focus group sessions consisting of 9, 5, 5, and 7 patients were conducted using a semistructured format focusing on the multidimensional concept of HRQL in LPR. Each focus group session lasted 2 hours and was audiorecorded for later analysis. Three facilitators including a senior gastroenterologist (H.-C. Lien), a senior otolaryngologist (C.-C. Wang), and an expert in group dynamics (S.-C. Chang) conducted the discussions. Each session began with a brief introduction, reiteration of the discussion purpose, discussion of rules, and assurance of confidentiality. Participants described their LPR symptoms, and how these discomforts impacted their daily life including physical, mental, and social well-being.

### 2.5 Collecting PRO data in clinical settings

Eligible patients filled out the PRO instruments including the GERDyzer,^[9]^ the RSI,^[5]^ and the ReQuest^[26]^ at baseline and underwent a 12-week esomeprazole treatment course. After a 2- to 4-week run-in period, esomeprazole 40 mg twice daily (Nexium; AstraZeneca Pharmaceuticals, Sweden) was prescribed because a twice daily dose was previously shown to be superior to a regular dose in a subset of patients.^[31]^ Adherence to treatment for 12 weeks, repeated PRO measurements, concomitant medication, and adverse events were evaluated at the 4th-, 8th-, and 12th-week follow-up visits.

### 2.6 Data analyses

#### 2.6.1 Focus groups

Two independent researchers (C.-P. Wu and S.-C. Chang) identified the common themes from the verbatim transcriptions of the 4 focus groups based on the concept of LPR-related HRQL. The identified themes were subsequently reconciled by the panel (H.-C. Lien, C.-P. Wu, and K. Chong) in an iterative process of data collection and analysis after the first focus group, which evolved during the analysis of data obtained from the 2nd to 4th focus group. Patients’ experiences unrelated to the concept of interest such as senile hearing loss were not included in the thematic analysis. We used a saturation table to assess and document saturation of concept elicitation from focus groups by emerging themes ordered by successive focus groups.^[32]^ Subsequently, the conceptual match was evaluated by mapping the themes derived from focus groups (universe of content) into the GERDyzer content (instrument content).^[29]^

To match patients’ responses with the instrument content, the relevance between the selected interview transcripts and identified common themes, and between the common themes and the dimensions of the GERDyzer, were assessed by 5 experts in the field of GERD or PRO (S.-C. Chang, W.-M. Liang, C.-C. Wang, S.-W. Lee, and C.-W. Ko) using CVI.^[23]^ An average I-CVI across all items >90% was considered excellent content validity.^[23]^ In addition, to ensure that the instrument captured the concept across the full range of the target population, we calculated the prevalence of each dimension of the GERDyzer by patient counts in all focus group participants, as well as in subgroups of different patient characteristics,^[29]^ including age (≥65 years vs <65 years), sex, symptom severity (baseline RSI total score >13 vs ≤13), and the concomitant typical GERD symptoms (presence vs absence).

### 2.7 Quantitative psychometric validation

#### 2.7.1 Floor and ceiling effects

The proportions of respondents with the lowest (0) and highest possible GERDyzer scores (10 for each item score, 70 for total score) were calculated for the presence of floor and ceiling effects. Levels of floor or ceiling effects <20% were considered adequate.^[33]^

#### 2.7.2 Reliability

Internal consistency and test-retest reliability of baseline data were calculated to assess the reliability.

*Internal consistency* was assessed by Cronbach α,^[34]^ ranging from 0 to 1. An acceptable Cronbach α value was between 0.7 and 0.9 in research,^[35]^ whereas values >0.90 may suggest redundancies in the scale.^[36]^

*Test-retest reliability* was evaluated by ICC in a random sample of 30 subjects with no evidence of change in clinical condition between 2 visits. An ICC value of >0.7 indicated a high degree of reliability.^[37]^ The second measurement of the GERDyzer scores was conducted 7 to 14 days apart from the baseline measurement in the same laboratory when they revisited to read the report of the examination performed in the previous visit.

#### 2.7.3 Construct validity

Construct validity includes structure validity, convergent validity, and discriminant validity.^[8]^

*Structural validity* was examined by means of confirmatory factor analysis (CFA) to determine whether the 2-factor model of the GERDyzer developed in the original validation study could fit LPR patients.^[9]^ A series of CFA using LISREL version 8.72 (Scientific Software International, Inc, Lincolnwood, IL) were performed to examine the structural validity. Model fits were considered acceptable, if the Bentler-Bonett normed fit index (NFI), non-normed fit index (NNFI), and comparative fit index (CFI) values exceed 0.9. The index standardized root mean square residual (SRMR) ≦0.08 was also used to evaluate the global model fit.^[38]^

*Convergent validity* was approached by correlating the GERDyzer and the validated scales instruments that evaluate related concepts including RSI and ReQuest at baseline. Pearson correlation coefficients with an absolute value of 0.4 to 0.7 reflect a moderately close correlation. Coefficients close to 1 indicate redundancy as they measure the same information, whereas coefficients near 0 indicate that the scales measure different concepts.^[39]^ An acceptable convergent validity was considered with coefficients >0.4.

*Discriminant validity* was quantified by constructing receiver-operating characteristic (ROC) curves and calculating areas under the curves (AUCs)^[40]^ to evaluate the ability of the GERDyzer to discriminate between 2 disease severity groups, that is, moderate-to-severe group (i.e., RSI >13) versus mild group (i.e., RSI ≤13) at baseline.^[5]^ This was based on the assumption that the disease severity was a major determinant of HRQL. An AUC value of >0.7 indicated a good discriminant validity.

#### 2.7.4 Responsiveness

The responsiveness to change during treatment was evaluated by effect size, which assessed the relative size of change. We divided the mean difference between baseline and week 12 by the standard deviation at baseline, and calculated effect sizes of total scale and each item. We also calculated effect sizes in subgroups of patients with or without concomitant typical reflux symptoms. An effect size of 0.2 was considered to be small, 0.5 to be medium, and 0.8 or greater to be large.^[41,42]^ The effective size >0.8 indicated a good responsiveness.

## 3 Results

### 3.1 Characteristics of participants

Twenty-six eligible subjects participated in 4 focus group discussions, and 100 eligible subjects were recruited from 347 consecutive subjects referred from otolaryngologists for quantitative psychometric validation (Supplementary Content 5). The mean age, sex, body mass index, and clinical characteristics were comparable among them (Table 1).

**Table 1:**
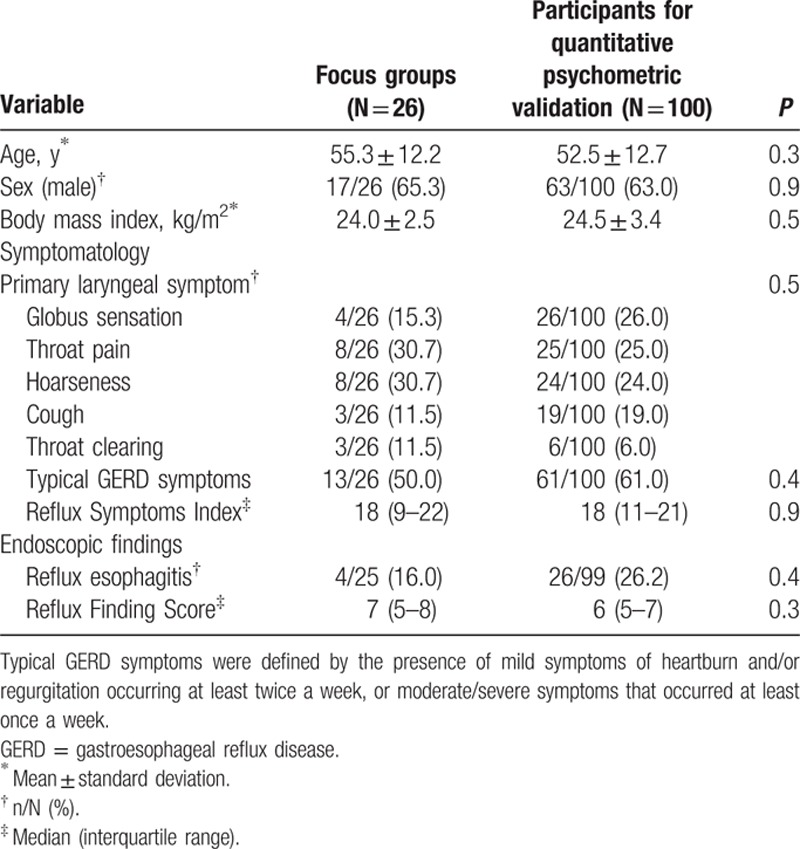
Demographic variables and baseline clinical characteristics of pH-test-proven laryngopharyngeal reflux patients compared between focus group and quantitative psychometric validation samples.

### 3.2 Translation of the GERDyzer

Cognitive interviews were documented in a summary including instruction, comprehension of each item, recall period, and response options. Using the item-tracking matrix, the committee discussed the cross-cultural adaptation, linking claims to concepts to items, and patient quotes. It was concluded that the Chinese version GERDyzer showed adequate linguistic adaptation and cultural harmonization. Moreover, no difficulties with respect to patient understanding were found, and no changes were required. All I-CVIs for evaluating the equivalence between Chinese version GERDyzer and the original version were 100% by 5 GERD experts.

### 3.3 Focus groups

Forty-four themes were identified from 674 selected interview transcripts related to the concept of interest in 4 focus groups. Saturation was demonstrated when no new information was elicited from the third focus group (Table 2). A strong conceptual match was shown by mapping 41 (93%) of 44 themes elicited from focus groups into the 10 dimensions of the GERDyzer content. The remaining 3 themes irrelevant to the GERDyzer content were related to the impact of adverse treatment effects such as concern about side effects of medications. Throat discomfort or voice problems adversely impacted role function, dietary habit, and emotion, particularly in the social and occupational settings (Table 3). The experience was consistent across different patient characteristics, including sex, age, disease severity, and status of concomitant typical GERD symptoms in subgroup analyses (Supplementary Content 6).

**Table 2:**
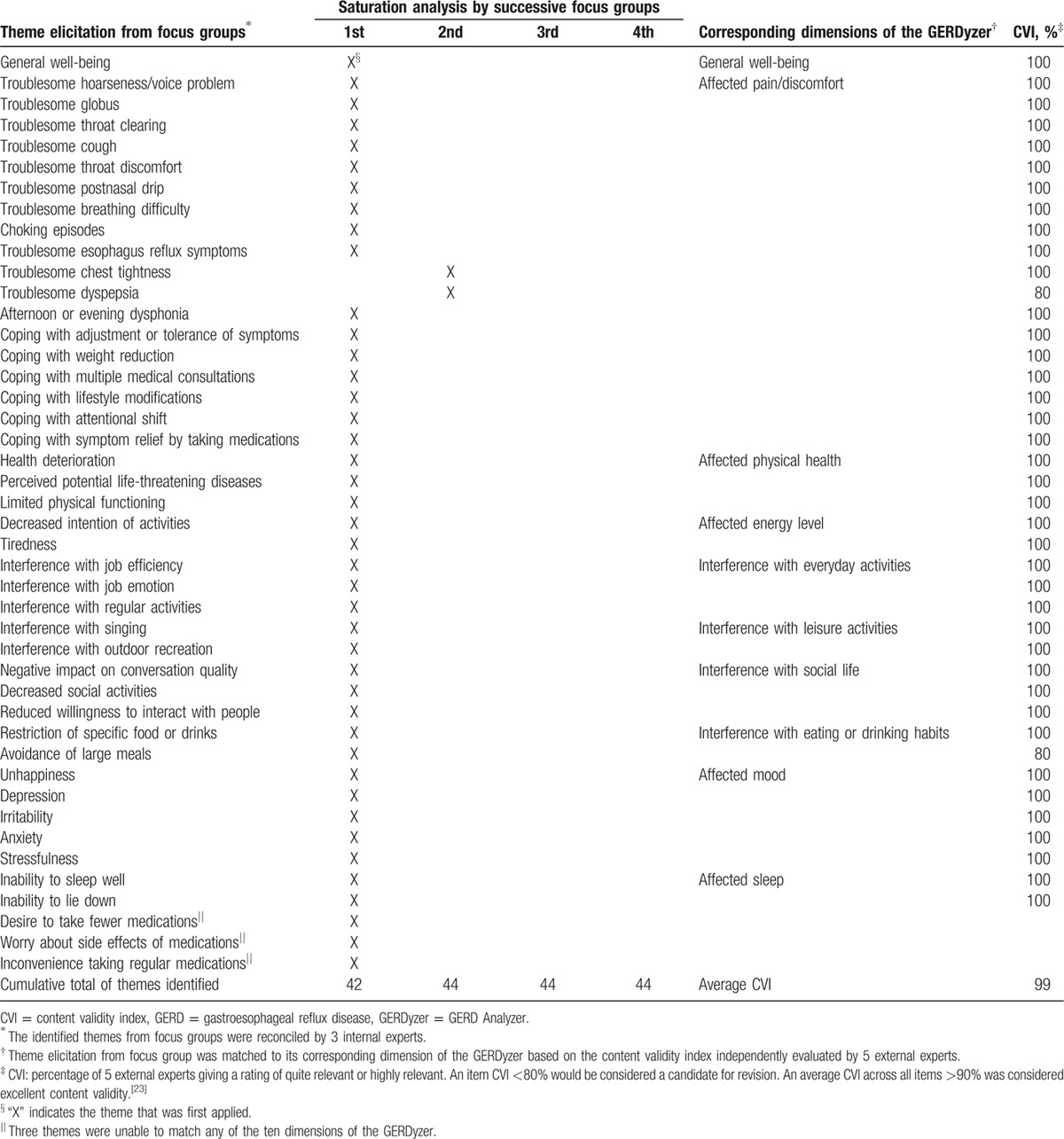
Theme elicitation from 4 focus groups and conceptual match with 10 dimensions of the GERDyzer.

**Table 3:**
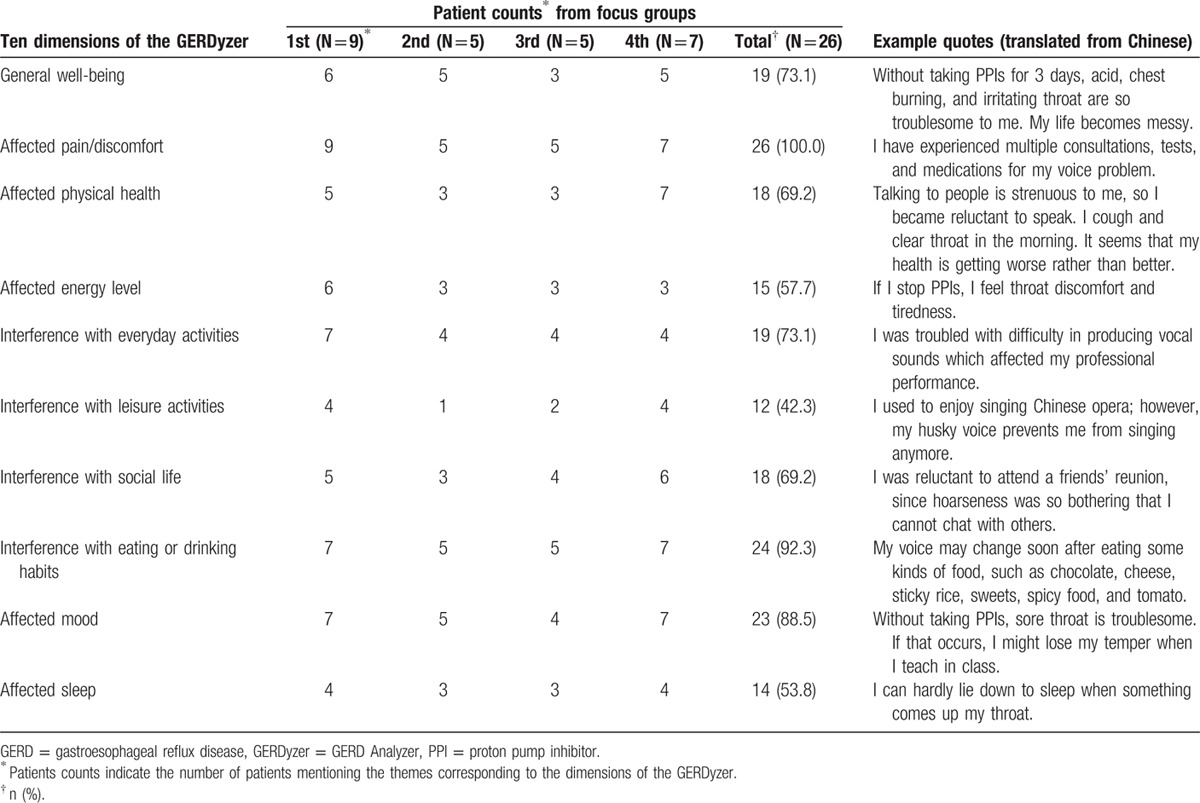
Patient counts and example quotes from focus groups of the 10 dimensions of the GERDyzer.

### 3.4 Quantitative psychometric validation

#### 3.4.1 Floor and ceiling effects

There were neither floor nor ceiling effects for each item score or for total score (Table 4).

**Table 4:**
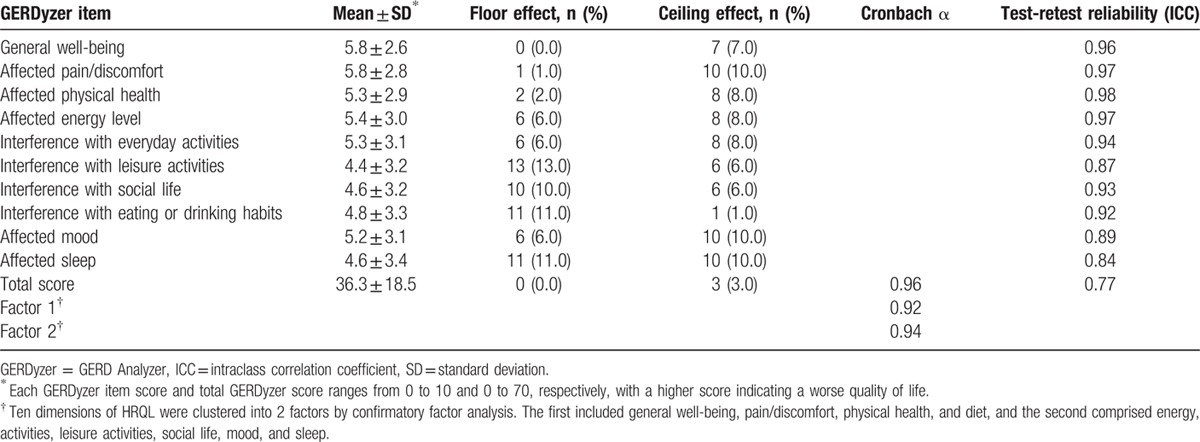
Score mean and standard deviation, floor and ceiling effects, reliability results, for baseline GERDyzer score.

### 3.5 Reliability

#### 3.5.1 Internal consistency

The Chinese version GERDyzer showed good internal consistency with a Cronbach α coefficient of 0.96 (Table 4).

*Test-retest reliability* was also good as demonstrated by an ICC of 0.84 to 0.98 (Table 4).

#### 3.5.2 Construct validity

*Structural validity* was corroborated by CFA. The model reached a good fit (NFI = 0.91, NNFI = 0.91, CFI = 0.93, SRMR = 0.05) when one pair of error variances was allowed to covary (i.e., “social life” and “mood”).

*Convergent validity* of the GERDyzer showed a logical correlation with the RSI, ReQuest total, ReQuest GI, and ReQuest-WSO (Table 5).

**Table 5:**
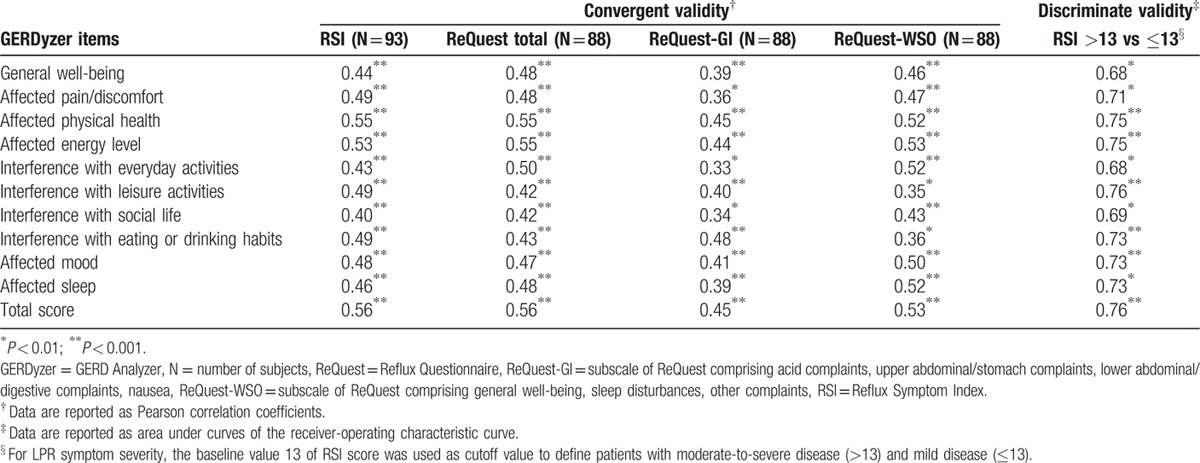
Convergent and discriminate validity of the GERDyzer scores.

*Discriminant validity* was supported by the ability of the GERDyzer to discriminate moderate-to-severe disease from mild disease at baseline (RSI >13 vs ≤13), with an AUC of the ROC 0.68 to 0.76 (Table 5).

#### 3.5.3 Responsiveness

Mean GERDyzer scores changed from 36.0 ± 18.2 at baseline to 17.0 ± 13.9 at week 12. The GERDyzer showed good responsiveness with all effect sizes >0.8 in LPR subjects, except for the dimension of affected sleep (0.75). In the subgroup analysis based on the presence or absence of concomitant typical GERD symptoms (Supplementary Content 7), the overall effect sizes were 1.20 and 1.21, respectively (Table 6).

**Table 6:**
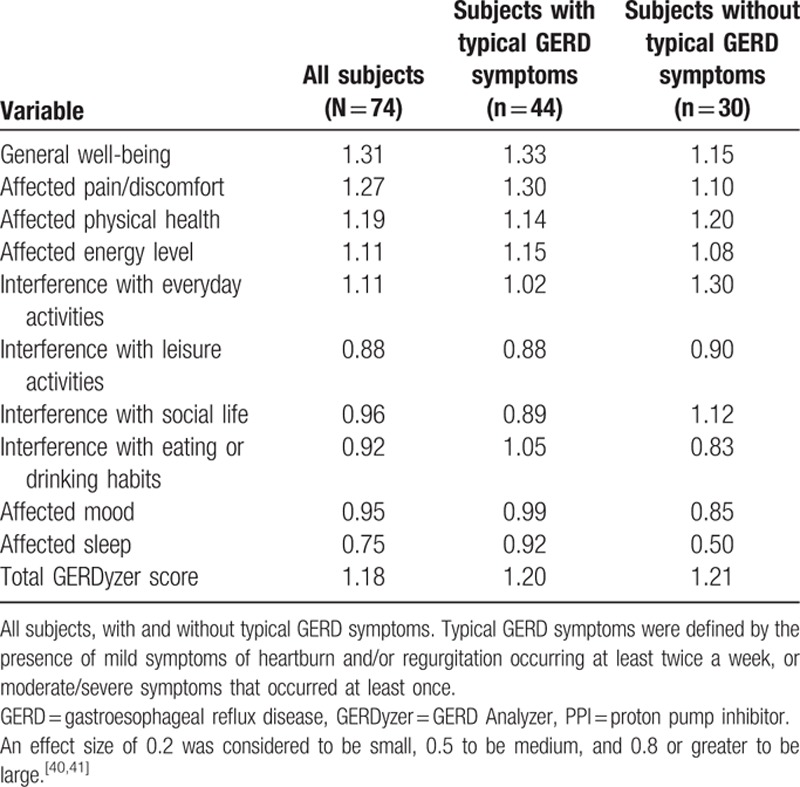
Effect sizes of the GERDyzer dimensions after 12-week PPI therapy in pH-test-proven laryngopharyngeal reflux patients.

## 4 Discussion

In this study, we cross-culturally adapted the GERDyzer into Chinese and explored the content validity of the GERDyzer in pH-test-proven LPR patients. We found a strong match between the GERDyzer contents and responses from the focus group participants. We also found that the psychometric properties of the Chinese version GERDyzer showed evidence of good reliability, validity, and responsiveness for LPR patients.

An ISPOR task force report addressed the principle of evaluating and documenting the use of existing PRO instruments.^[29]^ They emphasized the importance of the conceptual match between the PRO instrument and the intended claim using direct patient input such as focus groups from the target population. In the present study, we adopted saturation table procedures to assess and document the breadth of interview content in qualitative analyses.^[32]^ We found that saturation of concept elicitation was achieved and a strong conceptual match with the content of the GERDyzer was evident except for one dimension related to the impact of potential adverse effects of medications (Table 2). In the focus group discussions, the most commonly mentioned impact on HRQL was symptom-related pain or discomfort (100%), followed by dietary habit (92.3%), mood (88.5%), daily activity (73.1%), and general well-being (73.1%). In fact, 9 of 10 dimensions of the GERDyzer were endorsed by >50% of the focus group participants, although the dimension of interference with leisure activities was endorsed by only 42.3% of the focus group participants. Our results corroborate the findings of a previous focus group study conducted by Lenderking et al. They also found that physical well-being, role function, and emotional well-being were the main dimensions of HRQL impact in patients with LPR, particularly in the social and occupational settings.^[30]^

The Chinese version GERDyzer showed evidence of acceptable reliability, which was supported by the high internal consistency and high test-retest coefficients in this quantitative psychometric validation study. However, the value of Cronbach α coefficient was >0.9, as in the original English version, suggesting that some items may be redundant.^[43]^ As such, deleting items would result in a shortened version and reduce the time needed for its completion.^[44]^ Future research may be needed to explore this issue. Structural validity using the technique of factor analysis may confirm the clinical-empirical process. The 2-factor structure model developed in the English validation study in patients with GERD was also applicable in our patients with LPR, which may therefore support the weighting for total score calculation in the Chinese version GERDyzer. Two reference symptom-based PRO instruments were used to represent the concepts of LPR and GERD in this study: the RSI and the ReQuest, respectively. Moderate correlations between the Chinese version GERDyzer and the 2 instruments may support convergent validity. The magnitude of the RSI score using the cutoff point of 13 would dichotomize the patients into moderate-to-severe disease and mild disease. Based on the assumption that symptom severity is a major determinant of HRQL, 7 of 10 GERDyzer dimensions at baseline were able to discriminate moderate-to-severe disease from mild disease, as evidenced by area under the receiver-operating curves of >0.7, supporting the discriminant validity. Finally, the Chinese version GERDyzer was sensitive to change, as supported by the large effect sizes (>0.8) in total and in each of the dimensions regardless the presence or absence of concomitant typical GERD symptoms, except for the dimension of affected sleep (Table 6). The explanation for a smaller effect size (0.75) of sleep dimension might be due to predominant upright reflux in patients with LPR.^[45,46]^

Currently, the Laryngopharyngeal Reflux Health-Related Quality of Life (LPR-HRQL) developed by Carrau et al is the only available LPR-specific PRO instrument for evaluating HRQL.^[6]^ The LPR-HRQL is a 43-item instrument covering 4 adverse symptom-based domains, that is, hoarseness, cough, throat clearing, and swallowing, and another domain of “overall impact of acid reflux” which evaluates effects on multidimensional HRQL. An advantage of this instrument is that it is capable of identifying specific symptom-related impacts on HRQL. However, an advantage of the GERDyzer is that it can be used to obtain a single score for interpretation and for between-group comparison,^[8]^ if the patient only has one or more but not all laryngeal symptoms.^[7]^ Another difference between the 2 instruments is the interference with dietary habits, which was notably a common concern in our focus group participants, but did not seem to be a major issue in the LPR-HRQL.

The present study has 3 main strengths. First, the use of a saturation table to examine the completeness of response to HRQL themes in focus groups fulfilled the U.S. FDA guidance which recommends using an existing PRO instrument to assess various clinical conditions.^[12]^ This method allows transparent and auditable analyses for conceptual contents of the GERDyzer and patient inputs, which can then be compared with other qualitative research.^[32]^ Second, because the GERDyzer instrument uses the descriptor of “illness” rather than any specific reflux symptoms to describe the impact on HRQL,^[8]^ clinicians do not need to select either a GERD- or a LPR-specific PRO instrument, or use both simultaneously to measure HRQL in patients with both symptoms. Third, in response to the U.S. FDA guidance for PRO instruments in support of labeling claims for medical products,^[12]^ multidimensional HRQL PRO instruments may be easier to quantify compared with the symptom-based PRO instruments to determine a responder definition for the endpoint measure in patients with a disease exhibiting diverse symptoms such as LPR.

There were some limitations in this study. First, the diagnostic role of pH parameter used in this study has not been confirmed given the lack of a diagnostic gold standard for LPR. However, the severity of LPR symptoms was associated with the number of distal esophageal acidic reflux events in a physiological study,^[47]^ and abnormal esophageal pH was found in 81% of 128 patients with highly suspected LPR in a recent study, which could not be predicted from baseline presence or absence of typical GERD symptoms.^[46]^ We also recently demonstrated that in LPR patients without concomitant typical GERD symptoms the abnormal esophagopharyngeal pH was able to predict laryngeal symptom response to proton pump inhibitor (PPI) therapy.^[15]^ These findings may corroborate the diagnostic role of esophageal acid parameters and are consistent with the recent American College of Gastroenterology guidelines, which recommend pretreatment reflux monitoring in patients without concomitant typical GERD symptoms.^[48]^ Second, the study participants were recruited from a single referral center with a relatively small sample size. This limitation, however, can be attenuated in part by applying a reliable and responsive PRO instrument in a cohort receiving PPI therapy with a long follow-up period. Third, one may argue that the GERDyzer mimics a generic instrument from its face validity. However, each GERDyzer item contains a graph representative of the impact of a reflux event on each dimension of HRQL, indicating that it is a condition-specific instrument. It is also sensitive to changes during PPI treatment even in our patients without concomitant typical GERD symptoms. Fourth, treatment-related adverse effects were notably reported in the focus groups, which had a negative impact on HRQL from the patients’ perspective, but these could not be classified into any dimensions of the GERDyzer. Future development or modification of the HRQL PRO instruments of LPR may need to take this issue into consideration.

In conclusion, the content validity of the Chinese version GERDyzer was found to be adequate with linguistic and cultural adaptation in Taiwanese patients with LPR. It was also found to be reliable, valid, and responsive to change when applied in pH-test-proven LPR patients.

## Supplementary Material

Supplemental Digital Content
