# Supplementary material for: The suitability of the GERDyzer instrument in pH-test-proven laryngopharyngeal reflux patients
Source: Medicine (Baltimore). 2016 Aug 7;95(31):e4439. doi: 10.1097/MD.0000000000004439 (PMC4979824; doi:10.1097/MD.0000000000004439)
Supplement: Supplemental Digital Content [file medi-95-e4439-s001.doc]

Supplementary Content 1. Exclusion criteria.

1. respiratory or gastrointestinal malignancy
2. radiation therapy or surgery for the head, neck, lung, or gastrointestinal tract
3. trauma or surgery near the larynx
4. current or history of heavy smoking, substance or alcohol abuse history
5. infectious laryngitis in the previous 3 months
6. exposure to environmental irritants in the past 3 months
7. vocal cord papilloma, enlarged lingual or palatine tonsils, or goiters
8. excessive voice use
9. bronchial asthma
10. chronic cough attributable to angiotensin-converting enzyme inhibitor, or known chronic pulmonary or tracheobronchial etiologies, such as eosinophilic bronchitis, bronchiectasis, positive methacholine provocation test, or response to inhaled or systemic steroid
11. pharyngeal (Zenker’s) diverticulum or esophageal stasis syndrome, such as achalasia
12. chronic or allergic rhinosinusitis, nasal polyposis, or postnasal drip with response to at least 1 month of medical therapy with antihistamine, topical steroid spray, or defined by nasal endoscopy or computed tomography scan
13. participation in another investigational drug study in the previous month
14. acid suppressive therapy within 4 weeks prior to recruitment
15. need for continuous therapy with theophylline, iron supplements, warfarin, anti-fungal drugs, or digitalis
16. women were required to be non-pregnant and non-lactating and to maintain effective contraception if of child-bearing potential
17. a serious illness that would interfere with study participation, or refusal to participate
18. anxiety or depression with response to at least 1 month of an anxiolytic or an antidepressant
19. inability to fill out the questionnaire

Supplementary Content 2. Example of semi-structural probing questions for cognitive interviews to assess patient understanding and content coverage of the Chinese version GERDyzer.*

| Purpose | Probing | Participant Response |
| --- | --- | --- |
| Item stem: To understand the clarity of the question from the respondent’s perspective. | Can you tell me in your own words regarding Item #1: “How did you feel in general in the past 7 days”? What does this item content mean to you? | It’s asking how I felt and how bad the illness really was in general. |
|  | Can you describe any confusion or difficulty you had in understanding these instructions? | No, I did not have any confusion or difficulty. |
|  | Are there any words or phrases that you would change to improve the instructions? | No. |
| Recall: To identify how patients retrieve information, remember situations or events. | What period of time did you think about when you were completing the item? | The last seven days. |
|  | Describe your experiences with the concept over the timeframe. | For example, my irritating throat is so troublesome so that I couldn’t concentrate and do my job well. I also have a low mood, and couldn’t enjoy any recreation activities. |
| Response options: To understand how participants interpret the response options and make decisions around response choice. | Please read the response choice at each end of the horizontal line, i.e. “Excellent” and “Unbearably bad” and tell me what it means to you. | “Excellent” means that my illness did not give me any negative impact. “Unbearably bad” means that my illness was so troublesome to me that I could not tolerate it anymore. |
| Format: To identify the respondent's difficulties with the presentation of the questionnaire.) | What suggestions do you have for changing the format of the item so it is easier to complete? | None. This question is easy to complete. |

*This table demonstrates how we conducted cognitive interview in an example patient. The original results were in Chinese.

Supplementary Content 3. Example of a small section of a cognitive summary report*

| Cognitive interview summary | | | | |
| --- | --- | --- | --- | --- |
| Item presented in cognitive interviews | Subject responses to inquiry about what item means | Subject responses to inquiry about difficulty with item | Comments and discussion | Suggestion for changes to item (action to take) |
| Item#2: In the past 7 days, how much were you affected by pain/discomfort  resulting from your illness? | ID#1: In the past seven days, how severe was my experience of body pain or symptoms？  ID#2: It’s asking how uncomfortable I felt with the illness in the last week?  ID#3：It’s just trying to get me to rate the severity of pain.  ID#4：It’s asking how bad I was affected by the symptoms of the illness.  ID#5：When I read it, I was thinking how much pain I suffered due to the illness over the past 7 days.  ID#6：It’s asking how uncomfortable or painful I felt due to my illness in the last week.  ID#7：It’s asking me how bad or how severe my pain was.  ID#8 : How much painful sensation did I experience from my illness in the last 7 days.  ID#9 : It’s asking how bad I felt with my discomfort from the illness.  ID#10: To evaluate the severity of my pain or discomfort due to the illness.in the last week. | ID#1: No difficulty  ID#2: No difficulty  ID#3: No difficulty  ID#4: No difficulty  ID#5: No difficulty  ID#6: No difficulty  ID#7: No difficulty  ID#8: No difficulty  ID#9: No difficulty  ID#10: No difficulty | Patients did not have any confusion or difficulty in understanding the instruction. | Suggested change: None |

*For illustrative purposes only; cognitive summary reports may take various forms and the original results were written in Chinese.

Supplementary Content 4. The Chinese version GERDyzer instrument

| GERDyzer生活品質問卷 | | |
| --- | --- | --- |
| 您是患有食道逆流疾病的病患之一。這種疾病可能以各種方式對您的日常生活造成影響。我們希望透過本問卷來了解這種疾病是如何影響您的。  請以垂直線劃記最符合您的分數。 | | |
| 1. | 過去七天您整體感覺如何？ | 好極了-遭透了 |
| 2. | 過去七天，您受您的疾病所帶來的疼痛/不適感影響有多大？ | 完全沒有-非常嚴重 |
| 3. | 過去七天，您的疾病影響您的身體健康有多大？ | 完全沒有-非常嚴重 |
| 4. | 過去七天，您的疾病影響您的活力程度有多大？ | 完全沒有-非常嚴重 |
| 5. | 過去七天，您的疾病干擾您的每日活動有多大？ | 完全沒有-非常嚴重 |
| 6. | 過去七天，您的疾病干擾您的休閒活動有多大？ | 完全沒有-非常嚴重 |
| 7. | 過去七天，您的疾病干擾您的社交生活有多大？ | 完全沒有-非常嚴重 |
| 8. | 過去七天，您的疾病干擾您的飲食、吃與喝的習慣有多大？ | 完全沒有-非常嚴重 |
| 9. | 過去七天，您的疾病影響您的心情有多大？ | 完全沒有-非常嚴重 |
| 10. | 過去七天，您的疾病影響您的睡眠有多大？ | 完全沒有-非常嚴重 |

Supplementary Content 5. Figure illustrating enrollment of quantitative psychometric validation participants.


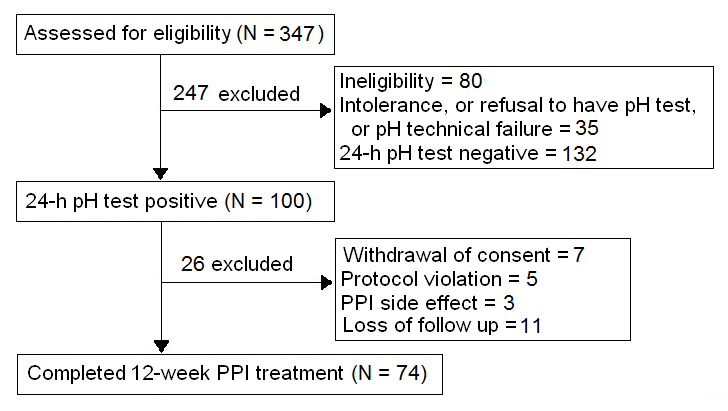


Supplementary Content 6. Figure illustrating the stratified analysis of participants by subgroups: (A) age, (B) gender, (C) symptom severity, and (D) status of concomitant typical GERD symptoms.


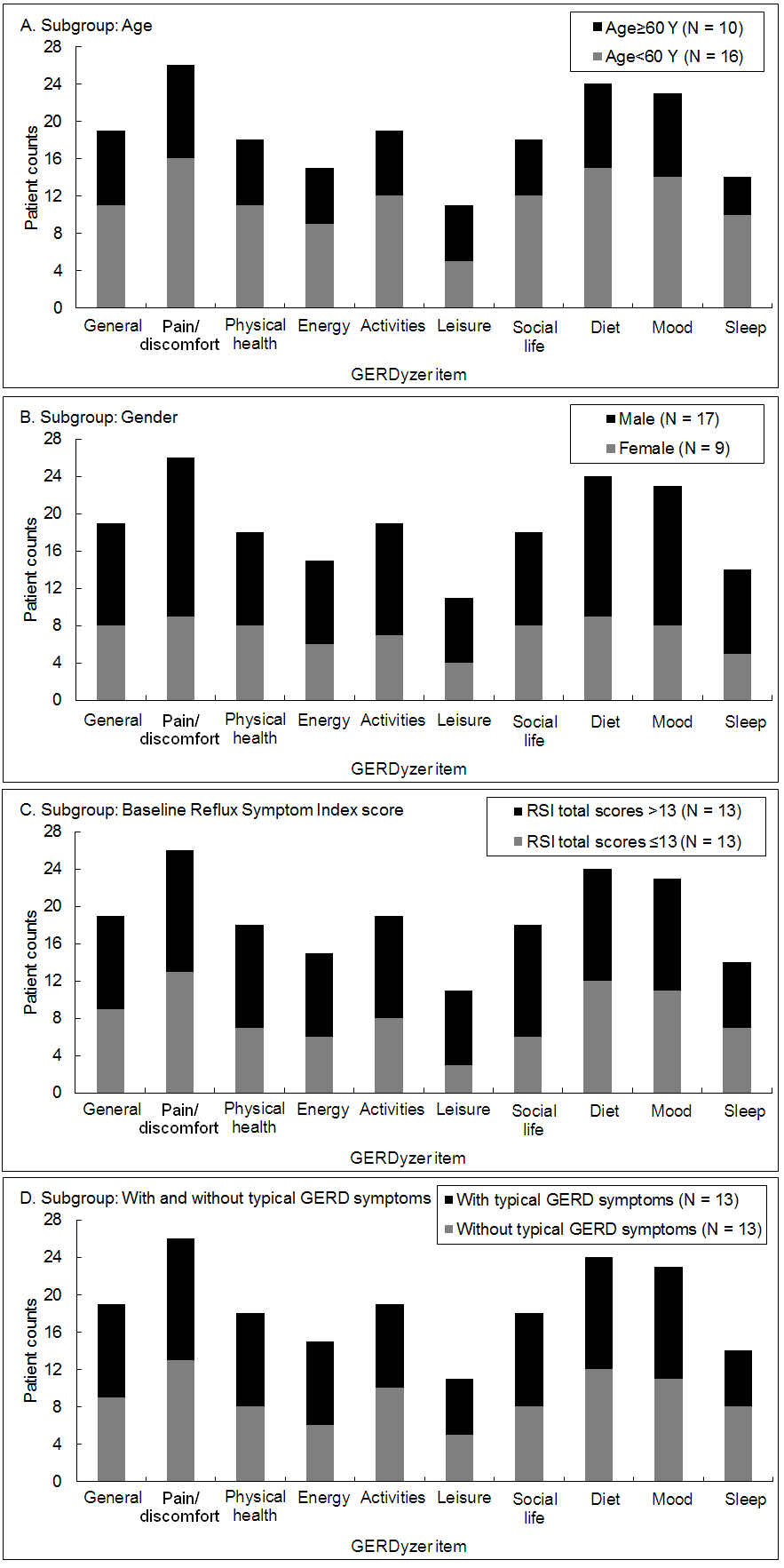


| Supplementary Content 7. Baseline Characteristics of the GERDyzer Dimensions in pH-test-proven Laryngopharyngeal Reflux Patients With and Without Typical GERD Symptoms | | | |
| --- | --- | --- | --- |
| Variable | With typical GERD symptoms  (n = 61) | Without typical GERD symptoms  (n = 39) | *P* |
| Age (years)^a^ | 52.0 ± 13.6 | 53.2 ± 11.3 | 0.6 |
| Gender (male)^b^ | 36/61 (59.0) | 27/39 (69.2) | 0.4 |
| Body mass index (kg/m^2^)^a^ | 25.0 ± 3.5 | 23.9 ± 3.0 | 0.1 |
|  |  |  |  |
| **Symptomatology** |  |  |  |
| Primary laryngeal symptom^b^ |  |  | 0.2 |
| Globus sensation | 18/61 (29.5) | 8/39 (20.5) |  |
| Throat pain | 15/61 (24.5) | 10/39 (25.6) |  |
| Hoarseness | 10/61 (16.3) | 14/39 (35.8) |  |
| Cough | 14/61 (22.9) | 5/39 (12.8) |  |
| Throat clearing | 4/61 (6.55) | 2/39 (5.12) |  |
|  |  |  |  |
| **Reflux Testing** |  |  |  |
| Reflux esophagitis^b^ | 16/61 (26.2) | 10/38 (26.3) | 0.9 |
| Reflux Finding Score^c^ | 6 (5, 7) | 6 (5, 7) | 0.7 |
| Pathological esophageal reflux | 51/61 (83.6) | 36/39 (92.3) | 0.2 |
| Pathological pharyngeal reflux | 27/61 (44.2) | 6/39 (15.3) | 0.006 |
| Typical GERD symptoms were defined by the presence of mild symptoms of heartburn and/or regurgitation occurring at least twice a week, or moderate/severe symptoms that occurred at least once a week.  Pathological esophageal reflux was defined as ≥4.6% of total acid exposure time with pH <4 at 5 cm above the upper margin of the lower esophageal sphincter.^1^  Pathological pharyngeal reflux was defined as ≥2 episodes of pharyngeal acid reflux.^1^  ^a^ mean ± standard deviation.  ^b^ n/N (%).  ^c^ median (interquartile range). | | | |

REFERENCES

Lien HC, Wang CC, Liang WM, et al. Composite pH predicts esomeprazole response in laryngopharyngeal reflux without typical reflux syndrome. Laryngoscope. 2013;123:1483-1489.
